# Supplementary material for: Association between oral microbiome and five types of respiratory infections: a two-sample Mendelian randomization study in east Asian population
Source: Front Microbiol. 2024 Apr 10;15:1392473. doi: 10.3389/fmicb.2024.1392473 (PMC11039966; doi:10.3389/fmicb.2024.1392473)
Supplement: Supplementary file 1 [file Data_Sheet_1.zip › Supplementary Materials/MR plots of saliva/Chronic sinusitis/s__Streptococcus_infantis_mgs_2056/leave_one_out.pdf]

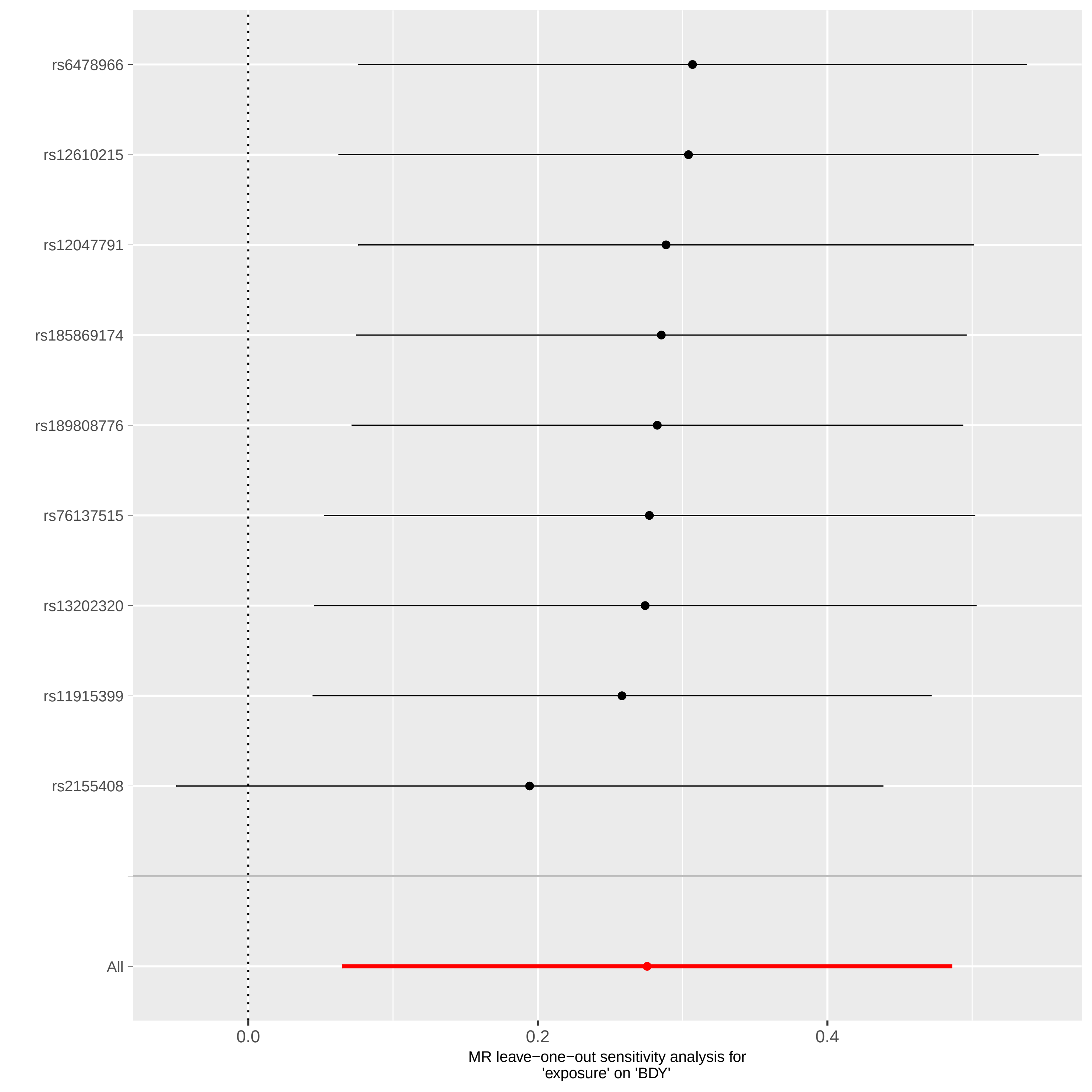
rs6478966
rs12610215
rs12047791
rs185869174
rs189808776
rs76137515
rs13202320
rs11915399
rs2155408
All
0.0
0.2
0.4
MR leave−one−out sensitivity analysis for
'exposure' on 'BDY'
